# Headache Associated with Sexual Activity—A Narrative Review of Literature

**DOI:** 10.3390/medicina57080735

**Published:** 2021-07-21

**Authors:** Piotr Ściślicki, Karolina Sztuba, Aleksandra Klimkowicz-Mrowiec, Agnieszka Gorzkowska

**Affiliations:** 1 Student’s Scientific Society, Department of Neurorehabilitation, Faculty of Medical Sciences in Katowice, Medical University of Silesia, Medyków 14, 40-752 Katowice, Poland; s73288@365.sum.edu.pl (P.Ś.); s75646@365.sum.edu.pl (K.S.); 2 Department of Internal Medicine and Gerontology, Faculty of Medicine, Jagiellonian University Medical College, Jakubowskiego 2, 30-688 Krakow, Poland; Aleksandra.Klimkowicz@mp.pl; 3 Department of Neurorehabilitation, Faculty of Medical Sciences in Katowice, Medical University of Silesia, Medyków 14, 40-752 Katowice, Poland

**Keywords:** headache associated with sexual activity, sexual headache, sexual cephalalgia

## Abstract

Headache associated with sexual activity (HAWSA) has accompanied humanity since ancient times. However, it is only since the 1970s that it has become the subject of more extensive and detailed scientific interest. The purpose of this review is to provide an overview of the development of the concept of HAWSA, its clinical presentation, etiopathogenesis, diagnosis and treatment especially from the research perspective of the last 20 years. Primary HAWSA is a benign condition, whose etiology is unknown; however, at the first occurrence of headache associated with sexual activity, it is necessary to exclude conditions that are usually immediately life-threatening. Migraine, hypnic headache or hemicrania continua have been reported to co-occur with HAWSA, but their common pathophysiologic basis is still unknown. Recent advances in the treatment of HAWSA include the introduction of topiramate, progesterone, and treatments such as greater occipital nerve injection, arterial embolization, and manual therapy. Whether these new therapeutic options will stand the test of time remains to be seen.

## 1. Introduction

The condition of headache associated with sexual activity (HAWSA) is hardly a new phenomenon. Headache associated with exercise or physical exertion has been noted as far back as classical Greece: Hippocrates is credited with the saying that one should be able to recognize those who have headaches from gymnastic exercises, or running, or walking or hunting, or any other unseasonable work, or from immoderate venery [1]. In medieval times the issue of HAWSA was the subject of Avicenna’s *Canon of Medicine* and traditional Persian medicine [2]. Initiated by Avicenna, the famous manuscript was written during the Islamic Golden Age (9th–12th century), then translated into Latin, and taught in medical schools up until 18th century. In the first chapter of the third volume of *Canon*, entitled *On the diseases of the head*, Avicenna discusses different kinds of headaches, including HAWSA. The headache was categorized into over twenty different types, and HAWSA was referred to as “headache associated with sexual intercourse,” which was a fairly modern term for that time [3]. 

“Headache associated with sexual activity” was included in the first edition of the 1988 International Classification of Headache Disorders (ICHD-1) [3], in the chapter: *Miscellaneous headaches unassociated with structural lesion*. In this classification, based on pain characteristics and presumed pathophysiologic mechanisms, this form of headache was divided into three types. The “dull type,” “explosive type,” and “postural type” of HAWSA were distinguished. Type one denoted a spasmodic-type headache with bilateral pressure pain that progressively increases with sexual arousal, type two was a vascular headache with sudden and throbbing pain occurring at the time of orgasm, and the last type, holo-cephalic, was a positional headache developing after sexual activity with clinical features consistent with low cerebrospinal fluid pressure headache and thought to be related to dura mater tear caused by intense sexual intercourse [4].

In ICHD-2 (of 2004), headache associated with sexual activity was included in Chapter IV as “Primary headache associated with sexual activity” and further subdivided into pre-orgasmic headache and orgasmic headache. These two types were directly related to the two first types in the 1988 classification. Postural headache was moved to the secondary headache disorders section, where it was classified as “Headache attributed to spontaneous (or idiopathic) low CSF pressure” [5].

The most recent and precise official document defining headache associated with sexual activity is the 2018 ICHD-3 [3]. Classification distinguishes between primary headache associated with sexual activity (4.3) and probable primary headache associated with sexual activity (4.3.1). According to this classification, primary headaches associated with sexual activity are a single entity with variable presentation and has replaced previously used terms such as: benign vascular sexual headache, coital cephalalgia, coital headache, intercourse headache, orgasmic cephalalgia, orgasmic headache or sexual headache. The previous division included into ICHD-1 and ICHD-2 into pre-orgasmic and orgasmic headache has been removed, since clinical studies were unable to distinguish this. In addition, new more specific diagnostic criteria have been introduced. 

According to the ICHD-3, diagnostic criteria for HAWSA include at least two episodes of pain in the head and/or neck fulfilling the following criteria: (1) the headache is brought on by and occurring only during sexual activity, (2) the pain in the head increases in intensity with increasing sexual arousal, and/or it has abrupt explosive intensity just before or with orgasm, (3) the pain lasts from one minute to 24 h with severe intensity and/or up to 72 h with mild intensity, (4) the pain cannot be better accounted for by another ICHD-3 diagnosis [3].

Probable primary headache associated with sexual activity can be diagnosed if patients experience only one attack of HAWSA during their lives and when the following criteria are met: (1) a single headache episode fulfilling criteria 2–3 or at least two headache episodes fulfilling criterion 2 and either but not both of criterion 3 and 4, (2) brought on by and occurring only during sexual activity, (3) either or both of the following: (3.1) increasing in intensity with increasing sexual excitement, (3.2) abrupt explosive intensity just before or with orgasm, (4) lasting from 1 min to 24 h with severe intensity and/or up to 72 h with mild intensity. The episode or episodes do not fulfill ICHD-3 criteria for any other headache disorder (5) and are not better accounted for by another ICHD-3 diagnosis (6) [3]. 

Epidemiological studies have shown that HAWSA occurs in 1–1.6% of the population [6,7] of all sexually active ages [8,9], more commonly in men—a male to female ratio of 1.2–4:1 [8,9], who are middle-aged, in poor physical shape, mild to moderately overweight and mild to moderately hypertensive [9]. The mean age of onset is 39.2 (±11.1) years, and it has two peaks: the first between 20 and 24 years of age, and the second between 35 and 44 years of age [10]. However, symptoms can occur at a very young age: a boy has been described who first experienced HAWSA at the age of 12 years [11]. It occurred with his first sexual experiences (masturbation, orgasm), suggesting that the headache was not the result of learning or behavioral conditioning. The patient also suffered from exertional headache, which is common in patients with HAWSA, and he had a family history of severe migraine, a headache often described as comorbid with HAWSA [11]. 

Headache associated with sexual activity can run in families. One study described four sisters suffering from this type of pain [12]. Another study described two cases of familial primary HAWSA suffered by a mother and daughter [13]. These reports suggest the existence of a genetic factor that could determine the familial occurrence of the condition. However, this question needs to be confirmed by further observations. 

Knowledge of HAWSA has expanded significantly in recent years, as reflected in the 2018 headache classification. Therefore, the purpose of this narrative review was to examine the literature to update the knowledge gained in the past 20 years regarding this rare disease entity. 

## 2. Methods

**Figure 1:**
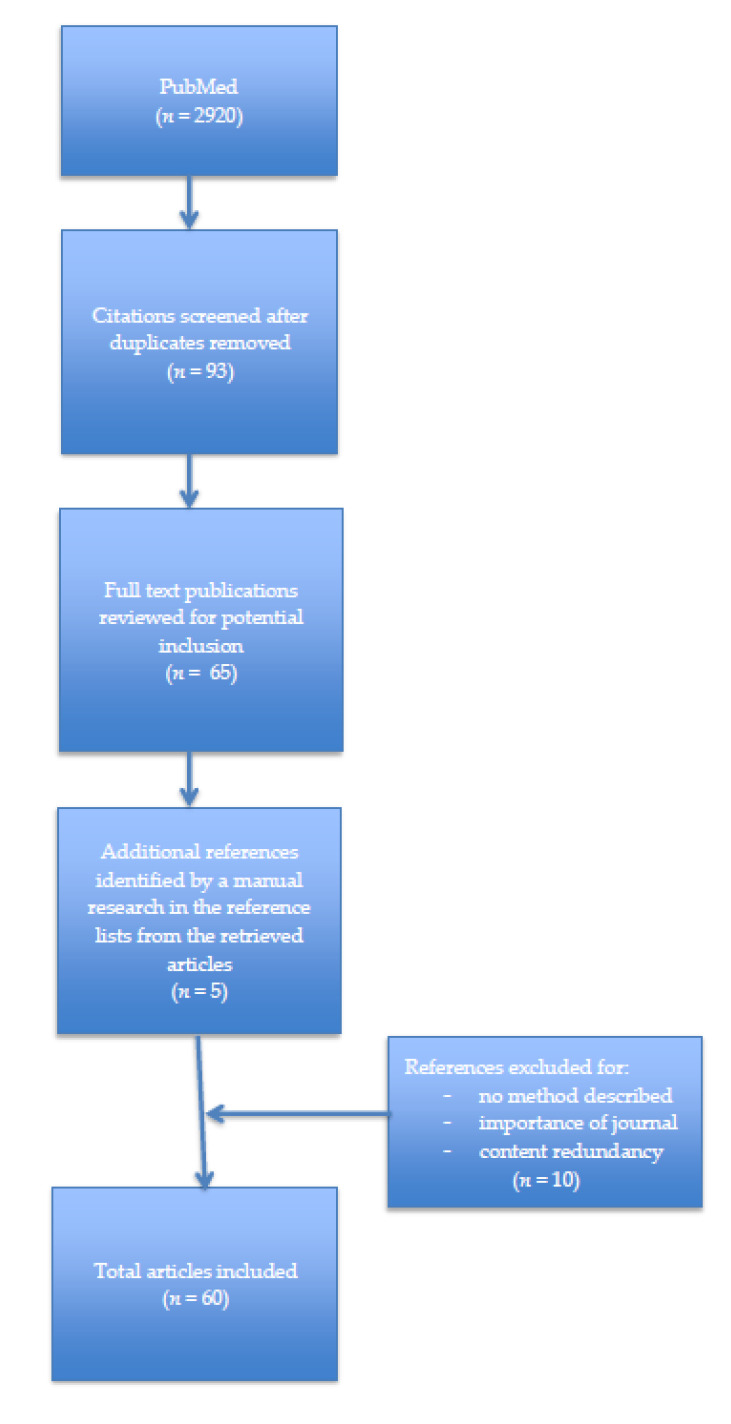
Flowchart of selection process.

A comprehensive literature search was conducted through PubMed from 1 January 2000 to 31 December 2020. The time frame of the last 20 years was chosen because it covers the period between the last two headache classifications and the additional recent years since ICHD-3, in which the new case reports on the clinical presentation, etiopathogenesis, diagnosis, and treatment of HAWSA have appeared and which were not included in the systematic review. In several cases, older sources that were particularly relevant to the topic were also cited. The following search terms: “sexual AND headache” (919 results), “coital AND headache” (27 results), “headache AND sexual activity” (322 results), “sexual AND cephalalgia” (799 results), “sexual AND cephalgia” (800 results), “coital AND cephalalgia” (26 results), “coital AND cephalgia” (27 results) were used and subsequently discussed by two researchers (P.Ś. and K.S.). A total of 2920 articles have been found, which were further sorted based on inclusion and exclusion criteria by other researchers (A.K.-M. and A.G.) and ultimately 60 studies were included. Figure 1. shows the flowchart of the selection process (Figure 1). A narrative analysis approach was used to interpret the results of the included studies. Because of heterogeneity and the scope of this review, no aggregate and statistical analyses were conducted. 

## 3. Clinical Picture

Headaches may occur during sexual activity related to intercourse or independent of intercourse (e.g., masturbation) or orgasm [14].

The duration of the headache varies from person to person. It can last as long as a few minutes, or as long as 2–24 h in more severe cases. In HAWSA of milder severity, it can last up to 72 h. The common denominator in all cases is intense pain during the first 5 to 15 min, then gradually decreasing. The duration of pain is usually longer in headache with onset during orgasm than in a headache preceding it. Nausea and phono- or photophobia are rather rare [5].

The pain is bilateral in two-thirds of patients, typically occipital or diffuse, and of a dull (47%), throbbing (47%), or stabbing (45%) quality [15].

It has been noted that sexual activity can sometimes be complicated by other neurological syndromes, such as amnesia [16]. Transient headache and amnesia associated with sexual activity are mild, self-limiting syndromes, probably of vascular origin.

## 4. Etiopathogenesis

The etiopathogenesis of HAWSA is unknown. It was hypothesized that it is a form of vascular headache. The mechanism of action might be mainly due to trigeminal–vascular effect with a distinct muscular component. In addition, some factors such as hypertension, pre-existing migraine, and psychological features are thought to be important in the development of HAWSA [9].

A number of determinants of HAWSA have been described. The majority of these are single case reports in which the authors search for an association between HAWSA and various etiologic factors; in many of these a clear cause-and-effect scenario can be identified, but there are also some in which the association is weak or questionable.

### 4.1. Medications and Stimulants

There is some evidence that drugs can have some effect on both the occurrence and intensity of HAWSA.

A brief report on the effects of amiodarone on HAWSA was published in 2002 [17]. The patient was a 52-year-old man who was prescribed amiodarone and metoprolol after mitral valvotomy surgery. Shortly thereafter, he presented with a sudden onset of severe throbbing pain appearing first in the left eye region and spreading to the right eye, lasting several minutes, without nausea or vomiting. In most cases, the headache was caused by sexual intercourse, but occasionally occurred after other physical activity. The patient quickly noticed that the headache had a strong association with amiodarone intake; the attacks increased in severity and frequency with increasing dosage and decreased with decreasing dosage. The authors hypothesized that the precipitating headache by amiodarone might be due to its vascular properties, in a similar mechanism to that of nitrate-induced headache.

Another study [18] reported a HAWSA caused by the use of ginkgo biloba glycosides. The patient used 19.2 mg twice daily for two weeks to enhance memory. On two occasions, a severe, bilateral, throbbing headache occurred during orgasm. The patient was discontinued from the medication and the symptoms resolved. The authors concluded that headache is one of the side effects of ginkgo glycosides.

The case of another patient who was a marijuana smoker and experienced the explosive type of headache associated with sexual activity is described [19]. He developed a posterior cerebral artery infarction during his first episode of orgasmic cephalalgia. He had a history of smoking up to 15 marijuana cigarettes per day for 9 years. Approximately 30 min before sexual intercourse, he smoked a marijuana cigarette. He then developed an explosive headache at the time of orgasm, which was immediately followed by visual disturbances in his left visual field and by left-sided paresthesias. In conclusion, the authors expressed the view that the effects of marijuana and orgasm on arterial blood pressure and cerebral autoregulation may have combined to induce transient contraction within the PCA and hypoperfusion in its vascular region.

### 4.2. Vascular Factors

In a study by Evers et al. 12 patients with the explosive type of HAWSA were studied during a headache-free state using an acetazolamide test and transcranial Doppler sonography [20]. The control group consisted of 12 age-matched migraine patients and 14 healthy subjects. The authors assessed changes in blood pressure, cerebral blood flow velocity (CBFV), and pulsatility index (PI). Patients with HAWSA showed significantly higher increases of blood pressure during standard physical exercise compared to controls. Changes of CBFV under exercise did not differ among the three study groups. After administration of 1 g of acetazolamide, CBFV showed a significantly greater increase in patients with HAWSA than in healthy subjects, and PI showed a significantly smaller decrease compared with healthy subjects and migraine patients. The authors concluded that these data suggest that the metabolic rather than myogenic component of brain vaso–neuronal coupling is damaged in patients with HAWSAs.

The purpose of a subsequent study was to describe the clinical characteristics of primary cough headache, primary exertional headache, and primary headache associated with sexual activity, and to evaluate the potential association with disorders of the cerebral or cervical venous circulation [21]. In conclusion, the authors found that headaches provoked by coughing and sexual activity may be related to disturbances in the venous circulation in patients. Venous stenosis may be considered a contributing factor to the onset of headaches.

### 4.3. Emotional State

In 2008 a case of a 44-year-old woman who was admitted to the hospital after three episodes of coital headache was presented [22]. Magnetic resonance imaging (MRI) and angiography (MRA) showed no vascular abnormality. The patient was discharged with a diagnosis of HAWSA. Despite abstaining from sexual activity, the patient experienced recurrent headaches several times a day with every change in her emotional state. The authors concluded that it was likely that the initial HAWSA triggered the mechanism that caused the recurrence, leaving the patient vulnerable to headache. Cerebral hemodynamic or neurochemical changes that occurred during sexual activity and would not normally cause pain could have easily triggered the headaches in patients in this headache-prone state. The exact cause of this state remained unknown.

### 4.4. Comorbidity of HAWSA with Other Headache

#### 4.4.1. Migraine

One case-control study of migraine patients examined the comorbidity of migraine and headache associated with sexual activity [23]. One hundred patients and 100 control subjects were asked about HAWSA. In five patients in the migraine group and none in the control group, a diagnosis of HAWSA could be established. Previous studies that had shown the co-occurrence of migraine and HAWSA have only included patients with HAWSA. Thus, the authors concluded that the association between migraine and HAWSA is bilateral.

Frese et al. conducted an interesting study on cognitive processing measured by event-related potentials (ERP) in patients suffering from the explosive type of headache associated with sexual activity [24]. In conclusion, the authors found that dysfunction in cortical information processing in HAWSA is very similar to that observed in migraine without aura. Since it is considered as a marker of vulnerability for both conditions, which argues for a pathophysiological link between the two and perhaps a common pathophysiological substrate. 

A peculiar case of a woman who complained of a migraine with aura attacks shortly after orgasm was described [25]. The short time period between orgasm and aura suggested a cause-and-effect relationship. The attacks began shortly after orgasm and were classified as type 2 of HAWSA. However, there were some differences: the patient spoke of twenty minutes visual phenomena, beginning two to five minutes after each orgasm, followed by mostly unilateral, throbbing pain. The authors concluded that their patient’s attacks of typical migraine with aura were probably triggered by orgasmic phase of sexual intercourse and activation of the limbic structures of the brain.

In 2018, young patients who experienced reversible orgasmic acephalgic symptoms meeting ICHD-3 criteria for migraine aura were reported [26]. The female presented with sensory and motor symptoms consistent with a brainstem aura, while the male patient presented with vertigo and typical visual aura. These symptoms occurred suddenly, without accompanying headache. There was no evidence of intracranial aneurysm or other structural pathology on additional examinations. The relationship between orgasm and aura was unclear. Both cases indicate that orgasmic migraine aura should be considered as part of the differential of sex-related neurological symptoms and clinically differentiated from fixed deficits, reversible cerebral vasoconstriction syndrome (RCVS) and post-orgasmic syndrome.

#### 4.4.2. Hypnic Headache

In 2009 a study by Porta-Etessam et al. presented the first report of the association between primary HAWSA and hypnic headache [27]. A 36-year-old man was referred to the headache clinic for recurrent headaches lasting for 3 months. It had an explosive onset, holo-cranial location, and was triggered exclusively by sexual activity, lasting for 5–30 min at a time. At the time of consultation, the patient had experienced more than 20 attacks. He also described another type of headache with episodes beginning one month after the initial presentation of the orgasm-related headache. It was qualitatively different, dull, of bilateral location, of moderate intensity, waking the patient, and lasting 3 h. According to the authors, the unusual association of headaches could be explained by randomness; however, on the other hand, both types of headache are relatively rare so they may reflect a common pathophysiological mechanism. Disturbed metabolic cerebrovascular autoregulation has been described as a factor responsible for primary HAWSA. The hypnic headache was associated with impairment of the suprachiasmatic nucleus function and may have cyclically triggered the mechanism leading to both sudden awakening and headache. In any case, common situational activation of the hypothalamic nucleus may have resulted in impaired cerebrovascular autoregulation and a suprachiasmatic nucleus function. Different studies on brain imaging have shown increased activation in the paraventricular nucleus of the hypothalamus during orgasm. Several cases are presented demonstrating the role of hypothalamus in the pathogenesis of vasospasm. The authors concluded that abnormal hypothalamus function might account for both types of headache, explaining the coexistence of both diseases in this patient.

#### 4.4.3. Hemicrania Continua

Prakash observed a patient with HAWSA and hemicrania continua [28]. It was difficult to determine, whether the occurrence of this rare association in the same patient was just a coincidence or whether they shared a common pathophysiology. The author emphasized that despite the different clinical characteristics of HAWSA and hemicrania continua, a common pathogenetic link between them has been speculated. The response of both headaches to indomethacin may suggest a common pathophysiology. Furthermore, hypothalamus activation has been observed in patients with hemicrania. Similarly, hypothalamic activation has been shown during sexual activity and orgasm.

## 5. Differential Diagnosis

Primary headache associated with sexual activity usually does not present with other abnormalities, e.g., disturbances in consciousness, vomiting, or visual, sensory or motor symptoms, whereas secondary HAWSA can do so [3]. When HAWSA first occurs, it is critical to exclude secondary causes of headache. The differential diagnosis of secondary HAWSA should include all potential causes of thunderclap headache, e.g., subarachnoid hemorrhage, arterial dissection, reversible cerebral vasoconstriction, cerebral ischemic or hemorrhagic stroke, and cerebral venous sinus thrombosis. Other pain syndromes that may mimic HAWSA include trigeminal neuralgia, demyelinating disease, cluster headache, migraine, and chronic paroxysmal hemi-cranias [4].

The most important diagnostic methods are magnetic resonance imaging (MRI), computed tomography of the brain (CT) and CT angiography (CTA). Lumbar puncture and CSF analysis may also be a useful diagnostic method.

### 5.1. Aneurysms

**Figure 2:**
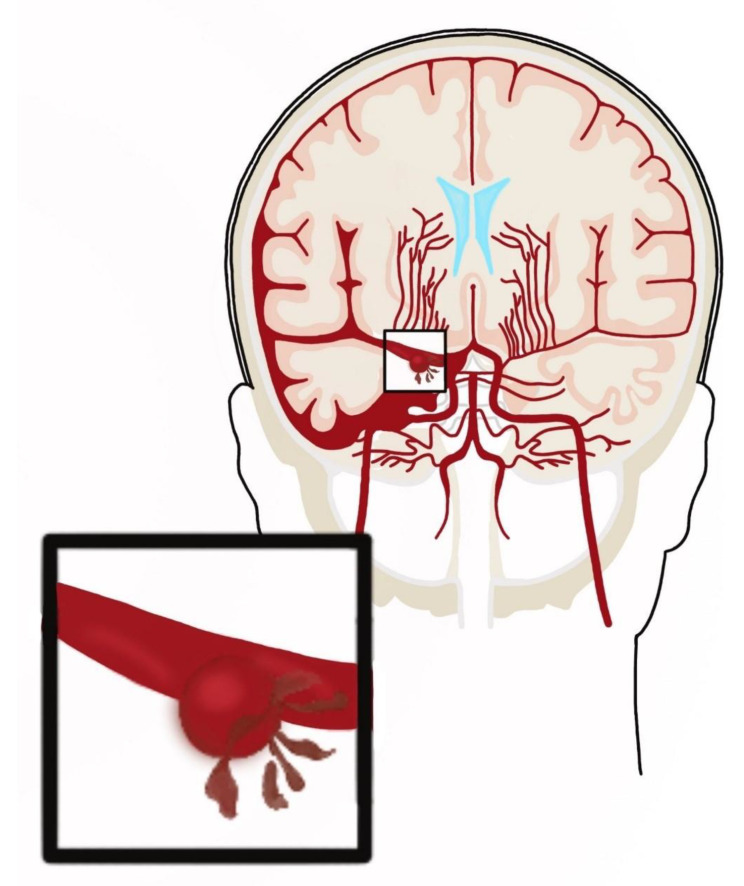
Aneurysmal subarachnoid hemorrhage.

**Figure 3:**
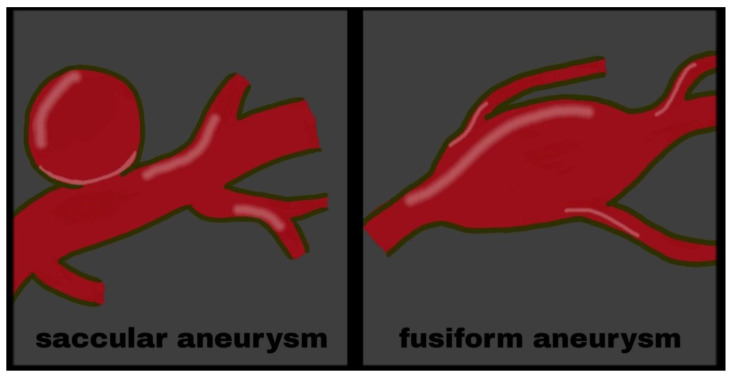
Aneurysms.

Multiple case series have reported that coitus was a preceding activity in 3.8–14.5% of patients with aneurysmal SAH, with males almost five times more likely to be involved [29]. Figure 2 shows the SAH from ruptured aneurysm (Figure 2). In addition, an unruptured aneurysm can cause headaches during intercourse and be a harbinger of SAH. Two patients with isolated recurrent coital/exertional headaches ipsilateral to unruptured fusiform aneurysms of the vertebral artery were described [30]. Another report described a 24-year-old man who was admitted to the emergency department for severe headache and vomiting after sexual intercourse [31]. He had a history of bilateral headache during orgasms in previous sexual activities. This time, however, he presented with a very severe headache, accompanied by nausea and vomiting. Diagnostics revealed the presence of subarachnoid hemorrhage from a saccular aneurysm. Figure 3 shows the schematic difference between a fusiform and saccular aneurysm (Figure 3).

### 5.2. Spontaneous Hemorrhage

A 61-year-old woman without comorbidities who presented to the emergency department with a HAWSA and vision loss was described [32]. She was diagnosed with an occipital and parietal intraparenchymal hemorrhage. It is likely that the hemorrhage was associated with an increase in intracranial pressure. This case demonstrated that cerebral hemorrhage could result from sexual intercourse, even without a predisposing cause.

Another case report described a 78-year-old man, referred to the stroke unit for sudden onset of a headache combined with speech and visual disturbances during sexual intercourse [33]. On admission, neurological examination revealed right-sided hemianopia with alexia. The authors suggested that the physical exertion during sexual intercourse might have been the cause of the intra-cerebral hemorrhage because there was a close temporal relationship between the physical exertion and the neurological symptoms. The authors argued that the severe jugular valve insufficiency along with exercise and forced exhalation might have facilitated the development of venous congestion and subsequent intra-cerebral hemorrhage. This was the first reported case of intra-cerebral hemorrhage during sexual intercourse in a subject with jugular valve incompetence.

### 5.3. Artery Dissection

A case of 48-year-old woman who noticed a slight headache on the top of her head thirty minutes after sexual intercourse was described [34]. Fifteen minutes later, she developed severe left-sided throbbing headache with visual disturbances, but no nausea, light, or noise sensitivity. She had a sudden sensation of warm fluid in her left eye and then vision in the lower part of the left eye field became dark, and within about 3 s vision in the entire field of the left eye became dark. The vision completely resolved after about 30–45 min. The day after the episode, a cerebral arteriogram revealed a left internal carotid artery dissection at the base of the skull. Another report was soon published on a 38-year-old man who presented to the emergency room with two episodes of orgasmic headache due to basilar artery dissection [35]. 

**Figure 4:**
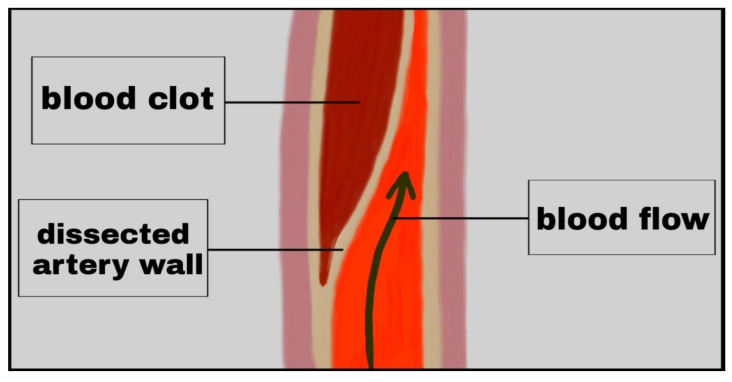
Arterial dissection.

A schematic drawing of a middle cerebral artery dissection is shown in Figure 4. 

### 5.4. Reversible Cerebral Vasoconstriction Syndrome

One common cause of a thunderclap headache is a reversible cerebral vasoconstriction syndrome (RCVS), often triggered by sexual activity.

In *Cerebral vasospasm and headache during sexual intercourse and masturbatory orgasms*, published in 2004 [36], Valença et al. studied the case of a 44-year-old woman who presented with both coital and masturbatory headaches during orgasm associated with “segmental reversible cerebral artery vasospasm.” Cerebral segmental vasoconstriction was visualized by magnetic resonance imaging and digital angiography before, during, and after resolution of clinical symptoms. In conclusion, the authors stated that the findings of cerebral artery narrowing, presented by some patients shortly after attacks of orgasmic headache, supported the hypothesis that segmental vasospasm may play a role in the pathogenesis of HAWSA.

Another study described the case of a 52-year-old man with a history of two episodes of coital headaches and recent cocaine, marijuana, and pseudoephedrine use, who presented with a sudden, sharp headache associated with photophobia and phonophobia [37]. The patient took two 60-mg pseudoephedrine tablets and boarded a plane. Shortly after the plane took off, the headache worsened, and the man started acting aggressively and arguing with the staff. He received intravenous fluids and diphenhydramine during the flight and was transported to the local hospital after landing. The next day, his headache did not resolve and he was diagnosed with posterior reversible encephalopathy syndrome (PRES) combined with reversible cerebral vasoconstriction syndrome (RCVS). After administration of nicardipine and subsequent switch to verapamil 180 mg daily, improvement in headache and normalization of blood pressure was achieved. The authors found that the patient had many risk factors for RCVS, such as a history of coital headache, cocaine, marijuana, and pseudoephedrine use, and being at a high altitude. The patient’s MRI showed only radiographic signs of PRES.

Reversible cerebral vasoconstriction syndrome is a poorly understood condition. The main hypothesis for the pathogenesis of RCVS indicates a transient deregulation of cerebral arterial tone, which may be triggered by sympathetic over-activity (e.g., sexual activity, physical exertion, emotions), endothelial dysfunction, and oxidative stress, and could lead to vasoconstriction [38].

It can be speculated that the relationship between HAWSA and RCVS may be bidirectional: RCVS is one of the many possible underlying causes of HAWSA, and on the other hand, patients with HAWSA may be more susceptible to developing RCVS, especially with exposure to vasoconstrictors.

Angiography in RCVS is abnormal, with alternating segments of arterial constriction and dilatations resembling sausages on a string; however, cerebral angiography, performed within the first week after headache onset, can be normal [38].

### 5.5. Spontaneous Intracranial Hypotension

A rare, often underdiagnosed cause of headache is spontaneous intracranial hypotension (SIH), which usually appears or worsens in the standing position and resolves in the supine position. A case is reported of a 28-year-old woman who developed a severe, unremitting, postural headache combined with photophobia immediately after reaching orgasm during sexual intercourse and immediately after assuming a standing position. The woman had undergone lumbar spine surgery for a herniated intervertebral disc 52 days before the onset of the headache. Neuroimaging revealed a dura mater tear, not recognized intraoperatively. Because the patient was asymptomatic for several weeks after surgery, and only developed symptoms while standing after sexual intercourse, the authors believe that the dural tear was probably the result of the physiologic stress of intercourse and orgasm, which caused an increase in intra-abdominal pressure and spinal fluid pressure [39].

## 6. Treatment

For patients who have been diagnosed with a primary headache during sexual activity, conservative, pharmacologic, or surgical management may be recommended. 

Conservative management consists of sexual restraint (usually for 3 months). Many patients can stop the headache or reduce its intensity by taking a more passive role during coitus [14].

Because of its relative rarity, pharmacological management of HAWSA is based on experience and case series, not on any randomized trials.

Some NSAIDs (ibuprofen, diclofenac), paracetamol, ASA, ergotamine, and benzodiazepines do not appear to have a beneficial effect when given before sexual activity [14]. Taking triptans before intercourse may be effective for short-term headache prevention with response rate of approximately 50% [40].

In patients with longer-lasting headaches or with recurring attacks, preventive treatment may be tried. Beta-blocker is usually indicated, propranolol [41,42], but also metoprolol or nadolol [43]. In patients who do not respond well to beta-blockers, indomethacin—non-steroidal anti-inflammatory drug, verapamil, flunarizine [42] or nimodipine [44]—calcium channel blockers, are recommended.

There are studies that show topiramate to be effective against HAWSA [45,46]. Topiramate is an anti-epileptic drug but it has also been shown to be effective for several other neurological and psychiatric conditions, such as migraine, cluster headache, diabetic neuropathy, and alcohol dependence.

For patients unresponsive to this prophylaxis and treatment, there are no clear future options.

In 2010, one case was described in which a man’s HAWSA was reported to be associated with his partner’s hormonal status [47]. A man had suffered from severe coital headache for 27 years. His condition rapidly improved in the first trimester of his partner’s pregnancy and the effects of the improvement lasted until the end of pregnancy. The condition worsened again after delivery and improved again when the partner had a second pregnancy. The authors speculate, that the probable mechanism of this beneficial effect was close cohabitation and absorption of progesterone through touch and smell. The patient was prescribed oral progesterone (norethisterone 5 mg)—one tablet around 30 min before sexual activity. The treatment was very successful with complete prevention of further attacks. Whether progesterone has an effect on HAWSA remains unclear at this time. This is the only such report to date.

Other treatments for HAWSA include invasive and non-invasive medical procedures.

Selekler et al. reported a 43-year-old man with explosive HAWSA [48]. The patient responded positively to a single injection of greater occipital nerve that included local anesthetic and steroid. The orgasmic headache resolved after treatment. The mechanism of action was not known. The authors suggested that because afferent impulses from the cervical muscles facilitated wind-up in C-fibers in the presence of dural inflammation, blockade of the greater occipital nerve might have reduced afferent movement from the periphery and relieved headache. Another theory presented was a modulation of nociceptive signaling in the central nervous system. However, the authors were unable to determine whether local anesthetic or steroid played a significant role in improving the patient’s condition. Although they could not rule out spontaneous resolution of symptoms, occipital nerve injection seemed to them to be a safe, inexpensive, and effective treatment for HAWSA with explosive quality.

In a recently published case-study [49], a case of a 19-year-old woman was reported, who presented to a primary care chiropractic clinic with intense, left-sided headache, occurring right before or during orgasm. The pain was diagnosed as primary headache associated with sexual activity by a hospital-based neurologist with experience in headache diagnostics. After seven sessions of manual therapy (lumbosacral spinal manipulative therapy), the patient reported remission of the HAWSA and the pain remained in remission at twelve months’ follow up. The authors concluded that the basis of the patient’s response to chiropractic therapy was unclear and speculative. According to the hypothesis presented in the paper, manual manipulation may have activated different central descending inhibitory pathways and may have stimulated neural inhibitory systems at different levels of the spinal cord. However, given the scientific evidence, the authors argued, that a definitive mechanism has not yet been found.

## 7. Conclusions

The exact pathophysiologic relationship between headache and sexual activity is still debated. HAWSA may be accompanied by other complaints, such as migraine, hypnic headache or hemicrania continua. There may be a common pathophysiological mechanism for these complaints, but this has not been identified to date.

A headache that occurs for the first time in connection with sexual activity always needs to be differentiated from symptomatic headaches. A history alone is usually not sufficient to make a diagnosis; additional testing is needed to rule out aneurysms, hemorrhages, arterial dissection, or reversible cerebral vasoconstriction syndrome.

Several substances have been shown in single reports to influence the occurrence of HAWSA. These include amiodarone, ginkgo biloba glycosides, and marijuana.

The most consistently reported responses in HAWSA are for indomethacin and beta-blockers (most commonly propranolol). Benefits have been reported with prophylactic use of topiramate and triptans, as well as triptans, in effectively reducing headaches preceding sexual activity.

Treatments such as greater occipital nerve injection or spinal manipulative therapy may be useful in achieving long-time remission.

Even if recent findings have not resulted in a significant breakthrough, they certainly make a fairly significant contribution to our knowledge of this rare but troublesome condition.

## Data Availability

Not applicable.
